# Treatment destinations and visit frequencies for patients seeking medical treatment overseas from the United Arab Emirates: results from Dubai Health Authority reporting during 2009–2016

**DOI:** 10.1186/s40794-019-0086-8

**Published:** 2019-07-02

**Authors:** Wafa K. Alnakhi, Jodi B. Segal, Kevin D. Frick, Altijani Hussin, Saifuddin Ahmed, Laura Morlock

**Affiliations:** 1 Department of Health Policy and Management Bloomberg School of Public Health, Baltimore, USA; 2 School of Medicine Johns Hopkins University, Baltimore, USA; 3 Carey Business School Johns Hopkins University, Baltimore, USA; 4 Economic Consultant, Baltimore, USA; 5 Department of Population, Family and Reproductive Health Bloomberg School of Public Health, Baltimore, USA

**Keywords:** Overseas treatment, Medical travel, Travel medicine, Public health, United Arab Emirates

## Abstract

**Background:**

Each year, the Dubai Health Authority (DHA) spends millions of dollars to cover the costs of United Arab Emirates (UAE) nationals seeking healthcare overseas. Patients may travel overseas to seek an array of treatments. It is important to analyze the number of trips and treatment destinations for patients travelling overseas to provide baseline information for the DHA to improve polices and strategies related to overseas treatment for UAE nationals.

**Methods:**

Administrative data were obtained from the DHA for UAE nationals who sought medical treatment overseas during 2009–2016. We examined the number of trips and treatment destinations by medical specialty, age, gender, years of travel and travel seasons. Multinomial logistic and negative binomial regression models were used to assess the relationships of the treatment destinations and number of trips, respectively, with the key variables of interest.

**Results:**

The study included data from 6557 UAE nationals. The top three treatment destinations were Germany (46%), the UK (19%) and Thailand (14%). The most common medical specialties were orthopedic surgery (13%), oncology (13%) and neurosurgery (10%). Oncology had the highest expected number of trips adjusted for a number of covariates (IRR 1.34, 95% CI: 1.24–1.44). Regarding destination variation, patients had a lower relative risk ratio of seeking healthcare in Germany in the winter (RRR 0.68, 95% CI: 0.57–0.80). Endocrinology was the most common medical specialty sought in the UK (RRR 3.36, 95% CI: 2.01–5.60).

**Conclusions:**

This is the first study to systematically examine the current practice of medical treatment overseas among UAE nationals. The results demonstrate that treatment destinations, medical specialties for which treatment was sought, age, gender and travel season are significant factors in understanding overseas travel for medical care. The study can guide the DHA in collecting more data for further research that may lead to policy-relevant information about sending patients to the best-quality treatment choices at an optimal cost.

## Introduction

The demand for global healthcare services is experiencing tremendous growth [1–7]. Each year, the Dubai Health Authority (DHA), the government health entity that oversees healthcare services in the emirate of Dubai, spends an average total expenditure of 77 million dollars to cover the costs for an average of 1500 UAE national patients seeking healthcare overseas [8]. The health sector in Dubai comprises government facilities, private facilities, and a free zone.^1^ The government of Dubai owns 4 DHA hospitals and 14 primary healthcare centers. In addition to the DHA, the Ministry of Health (MOH),^2^ owns and operates 2 hospitals and 9 primary healthcare centers in Dubai. The private sector comprises 22 hospitals and more than 1000 outpatient clinics and polyclinics [9]. Although the government of Dubai provides free healthcare services to UAE nationals as mandated by government law within government facilities, a number of patients travel to seek healthcare outside the UAE. Because there are many governmental entities in the UAE and in Dubai other than the DHA that sponsor UAE nationals for treatment overseas, the number of these patients is not accurately enumerated and cannot be easily traced.

Patients travelling overseas for healthcare seek an array of treatment options ranging from preventive procedures to complex surgeries, and they travel to different treatment destinations [10–13].The treatment destinations sought for healthcare are determined by patients and their families, often in consultations with physicians. As per the DHA decision (no.178) issued in 2009, any Emirati citizen, irrespective of socioeconomic status, is eligible to seek healthcare services overseas. Seeking treatment overseas is conditioned by the unavailability of treatment in the government sector or a better option overseas. The patients must provide an authenticated medical report from one of the DHA hospitals stating the unavailability of treatment in DHA hospitals or in the primary healthcare centers. The patient must sign and approve government rules and regulations for the treatment plan at the treatment destination and under the supervision of the DHA to be granted final approval.

Obtaining healthcare overseas may be associated with risks and complications compared with obtaining healthcare domestically [14]. Receiving routine follow-up treatment may also be challenging for many of these patients. Given the high cost of medical services overseas, the availability of free health services in the UAE and the potential for patient risks, it is important to explore and analyze the treatment destinations and the number of trips for overseas treatment [15–18]. This is the first study to provide baseline information for the government to improve polices and strategies related to seeking healthcare overseas.

## Methods

### Data source, study design, variables and measures

Administrative data were obtained from the DHA on UAE nationals who sought medical treatment overseas during the 2009–2016 period under the sponsorship of the DHA. The data contained the following information: birth date, gender, departure date, medical specialty sought overseas, and treatment destinations. The birth date was converted to age, with 7 groups as a categorical variable based on age-specific disease and medical specialty patterns by age. Gender was dichotomized into male and female. Departure date was used to create one categorical variable (travel season) and two continuous variables (number of trips and years in the study dataset). The number of trips variable was counted as a minimum of 1 trip to a maximum of 20 trips and defined as the number of trips taken by patients to treatment destinations. The medical specialty variable was a categorical variable with 42 medical conditions defined using the definitions of the American Board of Medical Specialties to improve standardization and increase precision. Medical specialty was defined as the specialty for which patients sought medical treatment at the destination. Patients who had more than one medical specialty reported in their record for a given trip (3.2%) were removed from the analysis. Treatment destination consisted of categorical variables and was defined as the treatment destinations to which patients traveled for medical diagnosis/treatment. Overseas treatment was defined in this study as the travel of patients from the UAE to treatment destinations for the purpose of legal diagnosis and treatment by UAE law, regardless of the level of complexity, under the sponsorship of the Dubai Health Authority. The shipment of laboratory samples or clinical results for diagnosis and clinical consultations as second options were excluded from the definition of overseas treatment. This study was limited to the period of 2009–2016.

### Ethical issues

The study protocol was submitted to the Johns Hopkins School of Public Health Institutional Review Board, where it was defined as nonhuman subjects research (IRB No: 00007896).

### Statistical analysis

The statistical analyses were conducted using Stata 13 (Stata Corporation, College Station TX). Means, standard deviations (SD), and Student’s t-tests were used for continuous variables. Frequency distributions, percentages, and chi-square tests were used for binary and categorical variables [19]. Two regression analysis models were constructed with 95% confidence intervals (CIs) and *p* < 0.05 indicating statistical significance [20, 21]. Our models were progressively adjusted for different sets of potential confounders. The first regression model was a multinomial logistic regression to estimate relative risk ratios (RRR) to identify factors associated with treatment destinations. The multinomial logistic regression model included age group, gender, travel season, and top 15 medical specialties variables. The Akaike information criterion (AIC) test was performed to choose the simplest model with the best fit; the model with the top 15 medical specialties out of 42 medical specialties had the lowest AIC, indicating the best fit. The second analytical model was a negative binomial regression to estimate incidence rate ratios (IRR) to identify factors associated with the number of trips. Our preliminary analysis suggested that the variance of the outcome was larger than the mean, and the likelihood-ratio test of alpha was significant, indicating the appropriate selection of the negative binomial model compared with a Poisson model [22]. The negative binomial model was adjusted for age group, gender, travel season, and top 15 medical specialties; years in the data set were used as the exposure period. Because the travel season and years in the data set variables were extracted from the same data field, namely, “the departure date”, the variance inflation factor (VIF) was used to assess collinearity; the mean VIF was less than 2, which indicated that there was no collinearity.

## Results

**Fig. 1:**
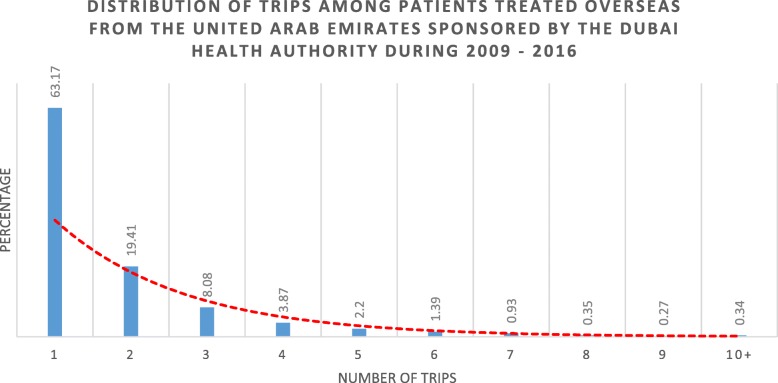
Distribution of trips among patients treated overseas from the United Arab Emirates sponsored by the Dubai Health Authority during 2009–20161

In total, 6557 patients from the UAE sought medical treatment overseas through the sponsorship of the Dubai Health Authority during 2009–2016. Among the age group category, patients aged 19–39 were the largest group (29%). The characteristics of the patients are shown in Table 1. The minimum and maximum number of trips for patients from the UAE who sought medical treatment overseas during 2009–2016 are shown in Table 2, and the distribution of trips is shown in Fig. 1. The patients treated overseas travelled to 24 destinations during their first trip. The predominant number of patients travelled to the Federal Republic of Germany (46%). The patients treated overseas travelled seeking treatment for 42 medical specialties. The top 3 medical specialties patients sought medical treatment for overseas during 2009–2016 based on the first trip were as follows: orthopedic surgery (13%), internal medicine oncology (13%), and neurosurgery (10%).

**Table 1:** Demographic characteristics for patients treated overseas from the United Arab Emirates through the Dubai Health Authority during 2009–2016 based on the first trip travelling to the top three treatment destinations

	Treatment Destination			
	Federal Republic of Germany N (%)	United Kingdom N (%)	Others*N (%)	*P* ***
Gender				0.044
Male	1605 (47)	624 (18)	1169 (34)	
Female	1424 (45)	654 (21)	1081 (34)	
Age Group				< 0.001
0–4 yrs.	235 (34)	330 (48)	126 (18)	
5–12 yrs.	215 (44)	139 (28)	138 (28)	
13–18 yrs.	168 (49)	87 (25)	88 (26)	
19–39 yrs.	920 (49)	344 (18)	609 (33)	
40–54 yrs.	630 (48)	187 (14)	490 (37)	
55–69 yrs.	565 (45)	128 (10)	572 (45)	
70+	296 (51)	63 (11)	227 (39)	
Medical Specialty				< 0.001
Orthopedic Surgery	536 (63)	91 (11)	219 (26)	
Internal Medicine: Oncology	261 (32)	116 (14)	448 (54)	
Neurosurgery	360 (57)	44 (7)	225 (36)	
Ophthalmology	56 (14)	82 (20)	275 (67)	
Neurology	239 (64)	56 (15)	77 (21)	
General Surgery	173 (51)	57 (17)	107 (32)	
Internal Medicine: Cardiology	160 (49)	72 (22)	93 (29)	
Obstetrics and Gynecology	124 (43)	79 (27)	88 (30)	
Unspecified Pediatrics	83 (33)	114 (46)	52 (21)	
Internal Medicine: Gastroenterology	116 (50)	47 (20)	67 (29)	
Urology	103 (52)	48 (24)	47 (24)	
Internal Medicine: Endocrinology	96 (55)	43 (24)	37 (21)	
Internal Medicine: Nephrology	69 (47)	16 (11)	62 (42)	
Not Specified Cases	11 (8)	15 (10)	117 (82)	
Unspecified Internal Medicine	54 (39)	19 (14)	67 (48)	
Others**	588 (48)	379 (31)	269 (22)	
Travel Season				< 0.001
Summer	857 (48)	352 (20)	582 (33)	
Fall	782 (48)	301 (18)	563 (34)	
Winter	578 (39)	301 (20)	597 (40)	
Spring	812 (49)	324 (20)	508 (31)	

*Thailand, United States of America, Kingdom of Spain, Republic of India, Republic of Singapore, Republic of Austria, Kingdom of Belgium, French Republic, Swiss Confederation, Arab Republic of Egypt, South Korea, People’s Republic of China, Republic of the Philippines, Kingdom of Saudi Arabia, Republic of Slovenia, The Hashemite Kingdom of Jordan, Czech Republic, Republic of Indonesia, Italian Republic, Kingdom of Morocco, Kingdom of Sweden, Republic of Turkey

**Internal Medicine: Pulmonology, Pediatrics: Surgery, Internal Medicine: Rheumatology, Plastic Surgery, Vascular Surgery, Physical Medicine and Rehabilitation, Dermatology, Screening & Check-up, Pediatrics: Oncology, Pediatrics: Nephrology, Pediatrics: Neurosurgery, Pediatrics: Gastroenterology, Pediatrics: Hematology, Pediatrics: Neonatology, Psychiatry, Pediatrics: Endocrinology, Oral & Maxillofacial Surgery, Internal Medicine: Infectious Diseases, Dental, Pediatrics: Pulmonology, Genetics, Pediatrics: Rheumatology

***Significance level *p* < 0.05

**Table 2:** Visit frequencies by demographic characteristics and medical specialty sought by patients treated overseas from the United Arab Emirates sponsored by the Dubai Health Authority during 2009–2016

	Minimum – Maximum Trips
Gender	
Male	(1–20)
Female	(1–18)
Age group	
0–4 yrs.	(1–16)
5–12 yrs.	(1–11)
13–18 yrs.	(1–10)
19–39 yrs.	(1–20)
40–54 yrs.	(1–13)
55–69 yrs.	(1–11)
70+	(1–10)
Medical Specialty	
Orthopedic Surgery	(1–20)
Internal Medicine: Oncology	(1–18)
Neurosurgery	(1–13)
Ophthalmology	(1–20)
Neurology	(1–11)
General Surgery	(1–11)
Internal Medicine: Cardiology	(1–12)
Obstetrics and Gynecology	(1–7)
Unspecified Pediatrics	(1–16)
Internal Medicine: Gastroenterology	(1–7)
Urology	(1–10)
Internal Medicine: Endocrinology	(1–7)
Internal Medicine: Nephrology	(1–20)
Not Specified Cases	(1–20)
Un Specified Internal Medicine	(1–11)
Others^a^	(1–16)
Travel Season	
Summer	(1–20)
Fall	(1–18)
Winter	(1–20)
Spring	(1–20)

^a^ Internal Medicine: Pulmonology, Pediatrics: Surgery, Internal Medicine: Rheumatology, Plastic Surgery, Vascular Surgery, Physical Medicine and Rehabilitation, Dermatology, Screening & Check-up, Pediatrics: Oncology, Pediatrics: Nephrology, Pediatrics: Neurosurgery, Pediatrics: Gastroenterology, Pediatrics: Hematology, Pediatrics: Neonatology, Psychiatry, Pediatrics: Endocrinology, Oral & Maxillofacial Surgery, Internal Medicine: Infectious Diseases, Dental, Pediatrics: Pulmonology, Genetics, Pediatrics: Rheumatology

### Associations with treatment destination

The multinomial logistic regression models examined factors associated with treatment destinations. Unadjusted and adjusted relative risk ratios (RRR) are shown in Table 3. The older age groups (40–70+) had lower relative risk ratios of seeking healthcare services in Germany compared to the youngest age group as a reference group. Patients treated overseas had a lower relative risk ratio of travelling to Germany in the winter (RRR 0.68, 95% CI: 0.57–0.80). On the other hand, all age groups had the lowest relative risk ratio of seeking healthcare in the UK. Males had a lower relative risk ratio of seeking healthcare in the UK compared with females (RRR 0.78, 95% CI: 0.67–0.91). The top 3 medical specialties for which patients from the UAE sought healthcare services in the UK were internal medicine endocrinology (RRR 3.36, 95% CI: 2.01–5.60), internal medicine cardiology (RRR 2.93, 95% CI: 1.95–4.40), and urology (RRR 2.93, 95% CI: 1.81–4.77).

**Table 3:** Unadjusted and adjusted relative risk ratios for travelling to Germany and UK compared with other treatment destinations as a reference group based on the first trip among patients treated overseas from the United Arab Emirates through Dubai Health Authority during 2009–2016

Independent Variables	Germany			UK		
	RRR*	95% CI	P-value	RRR*	95% CI	P-value
Age group						
0–4 yrs. old	1.00	–	–	1.00	–	–
5–12 yrs. old	0.76	(0.55, 1.06)	0.103	0.41	(0.29, 0.57)	< 0.001
13–18 yrs. old	1.02	(0.70, 1.50)	0.917	0.46	(0.31, 0.69)	< 0.001
19–39 yrs. old	0.78	(0.58, 1.05)	0.107	0.24	(0.18, 0.32)	< 0.001
40–54 yrs. old	0.68	(0.50, 0.92)	0.013	0.16	(0.12, 0.22)	< 0.001
55–69 yrs. old	0.58	(0.43, 0.79)	< 0.001	0.10	(0.07, 0.14)	< 0.001
70+ yrs. old	0.70	(0.50, 0.97)	0.031	0.11	(0.08, 0.17)	< 0.001
Gender						
Female	1.00	–	–	1.00		
Males	0.91	(0.81, 1.03)	0.134	0.78	(0.67, 0.91)	0.002
Travel Season						
Summer	1.00	–	–	1.00	–	–
Fall	1.04	(0.88, 1.22)	0.673	1.02	(0.83, 1.25)	0.857
Winter	0.68	(0.57, 0.80)	< 0.001	0.88	(0.72, 1.08)	0.234
Spring	1.12	(0.95, 1.32)	0.176	1.11	(0.91, 1.37)	0.309
Medical Specialty						
Orthopedic Surgery	1.00	–	–	1.00	–	–
Internal Medicine: Oncology	0.26	(0.20, 0.32)	< 0.001	0.87	(0.63, 1.22)	0.441
Neurosurgery	0.68	(0.54, 0.86)	< 0.001	0.57	(0.38, 0.86)	0.007
Ophthalmology	0.08	(0.06, 0.11)	< 0.001	0.57	(0.40, 0.82)	0.002
Neurology	1.28	(0.95, 1.73)	0.111	2.07	(1.34, 3.18)	< 0.001
General Surgery	0.67	(0.50, 0.90)	0.008	1.52	(1.01, 2.30)	0.047
Internal Medicine: Cardiology	0.77	(0.57, 1.04)	0.090	2.93	(1.95, 4.40)	< 0.001
Obstetrics and Gynecology	0.54	(0.39, 0.75)	< 0.001	2.18	(1.45, 3.28)	< 0.001
Unspecified Pediatrics	0.51	(0.33, 0.79)	0.002	1.49	(0.93, 2.36)	0.094
Internal Medicine: Gastroenterology	0.73	(0.52, 1.03)	0.069	2.14	(1.35, 3.37)	< 0.001
Urology	0.93	(0.64, 1.37)	0.729	2.93	(1.81, 4.77)	< 0.001
Internal Medicine: Endocrinology	1.08	(0.71, 1.63)	0.731	3.36	(2.01, 5.60)	< 0.001
Internal Medicine: Nephrology	0.48	(0.33, 0.71)	< 0.001	0.81	(0.43, 1.49)	0.493
Not Specified Cases	0.04	(0.02, 0.07)	< 0.001	0.31	(0.17, 0.57)	< 0.001
Un specified Internal Medicine	0.35	(0.24, 0.52)	< 0.001	0.92	(0.52, 1.64)	0.782
Other medical specialties	0.85	(0.68, 1.06)	0.139	2.20	(1.62, 2.99)	< 0.001

*Adjusted for age group, gender, travel season and medical specialty by using multinomial logistic regression as a model for analysis with other treatment destinations as a reference group

### Associations with the number of trips

The models examining factors associated with the number of trips were adjusted for the covariates of age, gender, travelling season, number of years present in the data set and medical specialty using a negative binomial regression. Unadjusted and adjusted incidence rate ratios (IRR) are shown in Table 4. The oldest age group of 70+ years had a lower incidence rate ratio for the expected number of trips (IRR 0.78, 95% CI: 0.71–0.86). Patients treated overseas had a higher incidence rate ratio of expected number of trips in the spring (IRR 1.08, 95% CI: 1.02–1.13) followed by the winter (IRR 1.07, 95% CI: 1.02–1.14). Patients had a higher expected number of trips with every additional year present in the data set (IRR 1.09, 95% CI: 1.08–1.09). Patients who sought treatment during their first trip for internal medicine oncology (IRR 1.34, 95% CI: 1.24, 1.44), ophthalmology (IRR 1.15, 95% CI: 1.05, 1.26), and general surgery (IRR 1.11, 95% CI: 1.01, 1.23) were likely to have more additional trips.

**Table 4:** Unadjusted and adjusted incidence rate ratios for the number of trips for patients treated overseas from the United Arab Emirates through the Dubai Health Authority during 2009–2016

Independent Variables	Unadjusted			Adjusted *		
	IRR	95% CI	P-value	IRR	95% CI	P-value
Age group						
0–4 yrs. old	1.00	–	–	1.00	–	–
5–12 yrs. old	1.00	(0.92, 1.09)	0.983	1.02	(0.94, 1.12)	0.634
13–18 yrs. old	0.91	(0.83, 1.01)	0.075	0.95	(0.86, 1.06)	0.379
19–39 yrs. old	0.89	(0.84, 0.95)	0.001	0.92	(0.85, 0.99)	0.031
40–54 yrs. old	0.95	(0.88, 1.02)	0.175	0.94	(0.87, 1.03)	0.170
55–69 yrs. old	0.91	(0.85, 0.98)	0.013	0.90	(0.82, 0.97)	0.009
70+ yrs. old	0.79	(0.72, 0.86)	< 0.001	0.78	(0.71, 0.86)	< 0.001
Gender						
Female	1.00	–	–	1.00	–	–
Male	0.98	(0.94, 1.01)	0.019	0.97	(0.94, 1.01)	0.186
Travel Season						
Summer	1.00	–	–	1.00	–	–
Fall	0.97	(0.92, 1.02)	0.270	1.00	(0.95, 1.06)	0.945
Winter	1.06	(1.00, 1.12)	0.033	1.07	(1.02, 1.14)	0.006
Spring	1.03	(0.98, 1.08)	0.288	1.08	(1.02, 1.13)	0.006
Years						
Year in the data set	1.09	(1.07, 1.10)	0.000	1.09	(1.08, 1.09)	< 0.001
Medical Specialty						
Orthopedic Surgery	1.00	–	–	1.00	–	–
Internal Medicine: Oncology	1.37	(1.27, 1.47)	< 0.001	1.34	(1.24, 1.44)	< 0.001
Neurosurgery	1.06	(0.98, 1.15)	0.151	1.07	(0.98, 1.16)	0.125
Ophthalmology	1.09	(0.99, 1.20)	0.069	1.15	(1.05, 1.26)	0.003
Neurology	0.97	(0.88, 1.08)	0.599	0.99	(0.90, 1.10)	0.898
General Surgery	1.03	(0.93, 1.14)	0.551	1.11	(1.01, 1.23)	0.039
Internal Medicine: Cardiology	1.02	(0.92, 1.13)	0.678	1.07	(0.96, 1.18)	0.221
Obstetrics and Gynecology	0.98	(0.88, 1.09)	0.700	1.01	(0.90, 1.12)	0.922
Unspecified Pediatrics	1.23	(1.10, 1.36)	< 0.001	1.10	(0.97, 1.24)	0.123
Internal Medicine: Gastroenterology	1.01	(0.90, 1.14)	0.812	1.06	(0.94, 1.19)	0.323
Urology	0.95	(0.84, 1.08)	0.448	0.97	(0.85, 1.10)	0.625
Internal Medicine: Endocrinology	0.93	(0.81, 1.07)	0.301	0.96	(0.84, 1.10)	0.531
Internal Medicine: Nephrology	0.99	(0.86, 1.14)	0.842	0.99	(0.86, 1.14)	0.916
Not Specified Cases	1.04	(0.91, 1.20)	0.555	1.10	(0.95, 1.26)	0.198
Unspecified Internal Medicine	1.06	(0.92, 1.22)	0.443	1.09	(0.95, 1.25)	0.227
Other medical Specialties	1.10	(1.03, 1.18)	0.007	1.05	(0.978, 1.1)	0.196

*Adjusted for age group, gender, travel season, years, and medical specialty using negative binomial as a model for analysis

## Discussion

Approximately half of the patients from the UAE who travelled overseas during 2009–2016 through the sponsorship of the DHA sought medical treatment in Germany during their first trip. Patients’ age and gender were sensitive to the treatment destination. Patients who travelled to Germany were more likely to seek medical treatment for orthopedic surgery during their first trip and more likely to travel in the summer season. Patients who travelled to the UK were more likely to seek medical treatment for internal medicine endocrinology, internal medicine cardiology and urology. On the other hand, patients who travelled for internal medicine oncology, ophthalmology and general surgery had a higher expected number of trips compared with patients who travelled for other medical specialties. The number of trips was more likely to increase in the winter and spring seasons. Moreover, the older the patient, the lower the number of future expected trips overseas.

Noncommunicable diseases are considered new public health challenges in the UAE [23–26]. Cardiovascular diseases, injuries, cancers, respiratory disorders, and cerebrovascular diseases are the most common public health concerns in the country. The vast majority of the patients sought care for these causes. Furthermore, the medical specialties in this research were recorded by their general names and not by ICD codes, which reduced the precision of knowing the exact name of the disease.

Studies on the association between age or gender and treatment destinations are limited [27–32]. Some studies have suggested that the sources of information are important factors in shaping patients’ decisions before going through the medical travel experience [33–37]. Many patients may use different sources of information to learn more about the treatment destination, physicians’ credentials and hospital reputation. Scholarly sources, media sources, and word of mouth are common methods used to seek information about medical travel experiences. In collective society cultures such as the UAE, people tend to lean towards recommendations through personal contacts, such as word of mouth, compared with other sources of information that are more likely to be used in individualistic societies [38]. More qualitative studies are needed to understand people’s perceptions, motivations, and reasons for seeking treatment overseas and choosing treatment destinations. In terms of the relationship between age and medical travel, studies suggest that younger adults are more likely to engage in medical travel than older people. This pattern is consistent with our findings because the 19–39 age group had the highest number of trips compared with older ages.

Our results indicate that orthopedic surgery was the most frequent medical specialty for which people travelled overseas during their first trip. There is a lack of studies in the UAE that address orthopedic or spine surgeries [39]. However, some studies have been conducted on rheumatoid arthritis in the UAE. Patients have been found to have delayed diagnosis and low disease-modifying anti-rheumatic drug (DMARD) utilization. Studies have illustrated a gap between the onset of the disease and timely referral to appropriate treatment options. Other studies have indicated that this lag time is due to many reasons, including a lack of public knowledge and awareness about rheumatoid arthritis as well as an imbalanced ratio of trained rheumatologists to the population. Hence, it is important to close these gaps by ensuring appropriate staffing levels per population and increasing public health awareness and educational campaigns through media and patient support groups. This will lead to increased access for patients and early detection, resulting in early intervention and better responses to treatment and patient outcomes [40–44]. These steps may reduce the need for overseas orthopedic treatment.

Internal medicine oncology had the highest expected number of trips overseas. Due to the lack of ICD codes in our available data, it was not possible to detect the variation of cancer types among gender and age groups in the study. In general, there are an insufficient number of clinical and pathological studies about cancer in terms of patterns and incidence rate reporting in the UAE [45–47]. According to the UAE - National Cancer Registry report for 2014 from the Ministry of Health, the incidence rate of cancer is 42 cases per 100,000, including both UAE nationals and non-UAE nationals. The most common cancers according to the report are C50 Breast for females and C18-C21 Colorectal for males. Pediatric cases aged 0–14 are more likely to be diagnosed with C91-C95 leukemia. Moreover, the report illustrated the distributions of malignant cases by age group in the UAE and showed that the age group of 55–59 years had the highest frequency of cancer, which mirrors our results because the age group of 40–54 years had the highest frequency of seeking treatment overseas for cancer followed by the age group of 55–69 years [48]. Another study in the UAE reported similar findings, indicating that the most common sites of malignancy were cancers of the gastrointestinal system in males followed by breast cancer in females [49].

Ophthalmology was another medical specialty with a high expected number of trips overseas. Previous studies conducted in the UAE linked lifestyle changes with diabetes mellitus and retinopathy [50–53]. Currently, the prevalence of diabetes in the UAE is among the highest in the world, with some estimates putting it in the top 5 countries [54]. This finding implies that the disease, especially when it is associated with other chronic conditions and complications such as retinopathy, might contribute to a sizable healthcare burden to the UAE population regarding ophthalmology. Therefore, early screening and diagnosis may prevent long-term complications. Patient education, a healthy diet, physical activity, and timely referral to treatment may reduce the chances of diabetes complications. Notably, complications of diabetes could be one cause of seeking treatment overseas for ophthalmology, and other factors and diseases could be involved.

It is important to acknowledge some limitations of our study. The data collected from the DHA did not include ICD codes or the severity of the disease, which could affect the precision of the medical specialty variable. However, the American Medical Specialty Board classification was used in an effort to achieve some standardization in data management. In addition, patients who had more than one medical specialty reported in their record for a given trip (3.2%) were excluded from our analysis because we assumed that including them could potentially introduce bias to the analysis via two mechanisms: we were unable to access these patients’ records for more information, and we were subsequently unable to know the primary medical specialty for which the patient travelled. Another limitation involves administrative data; we had limited access to many variables of interest that could better explain our modeling, such as the socioeconomic status of the patients, treatment expenses, and information about the treatment destinations. While the study was limited to patients sponsored through the DHA, we cannot generalize the results because the data cannot represent all patients who travelled under the sponsorship of other health authorities in the UAE. However, the availability of the data at the DHA is considered a strength. Additionally, the data can be used in the future to conduct longitudinal analysis to better understand changes in the patterns of overseas treatment related to treatment destinations and medical specialties.

## Conclusion

In conclusion, our study is one of the more comprehensive studies related to medical travel and therefore contributes to the limited empirical research in this field. The results demonstrated that treatment destinations, medical specialties for which treatment was sought, age, gender and travel season are significant factors in understanding overseas travel for medical care. Creating an overseas treatment registry system in the UAE would be an important step to capture all medical travelers sponsored by different government authorities [55, 56]. Establishing a registry that contains all the essential variables, such as patients’ demographics, ICD codes, and treatment details, including costs, would prepare the government to conduct future comparative effectiveness research that may lead to policy-relevant information about sending patients to destinations for lower cost and high-quality patient outcomes [57–60]. In addition, this strategy would directly influence and promote patients’ informed decisions when choosing treatment destinations for the necessary procedures.

Resources saved as a result of comparative effectiveness research can be allocated towards prevention measures for the most common noncommunicable diseases and can provide treatment options in the UAE, whether in the government or in the private sector [61–65]. The results of this study can also provide an evidence base to create a “follow-up care program” for patients who received treatment overseas, especially those with repeated trips [66, 67]. The follow-up care would help in increasing the chance of patients’ survival, improving patients’ quality of life, and assessing patients’ overseas experience and could provide a substitute that allows patients to stay in the country. This type of program could lead to the reduction of complications and risks associated with treatment overseas.

## Data Availability

The data that support the findings of this study are available from the Dubai Health Authority. However, restrictions apply to the availability of these data, which were used under special agreement for the current study; thus, these data are not publicly available. Data are available from the corresponding author upon reasonable request and with permission of the Dubai Health Authority.

## Footnotes

1 Free-trade zones (FTZs) are special economic zones established with the objective of offering tax concessions and customs duty benefits to expatriate investors.

2 The federal health authority which is responsible for the implementation of health care policy in all areas of technical, material, and coordination with the Ministries of State, and cooperation with the private sector in health locally and internationally.
